# Internet use and youth self-reported health over the course of COVID-19 in China

**DOI:** 10.3389/fpsyt.2025.1620416

**Published:** 2025-10-08

**Authors:** Ran Xie, Yang Cao, Wenbin Wang

**Affiliations:** Department of Sociology, Jilin University, Changchun, China

**Keywords:** internet use, lifestyle, interpersonal interaction, self-reported health, youth

## Abstract

**Introduction:**

The reshaping of lifestyles and interpersonal interactions by Internet use has brought about a networked characterization of youth self-reported health. Will the networked characterizations of youth's self-reported health change under the influence of the COVID-19?

**Methods:**

We used multiple linear regression models to test the hypothesis of moderating effect and the hypothesis of heterogeneity by analyzing data from the 2017 and 2021 China General Social Surveys.

**Results:**

First, Internet use can improve youth self-reported health by promoting healthy lifestyles and interpersonal interactions. Second, under the influence of COVID-19, the positive effect of Internet use on youth self-reported health through healthy lifestyles was weakened, but the positive effect of Internet use on youth self-reported health through interpersonal interactions was strengthened. Third, the information support of Internet use in terms of lifestyles and the emotional support in terms of interpersonal interactions have different enhancing impacts on youth self-reported health, i.e., high-frequency Internet use significantly promotes youth self-reported health, and this positive effect is more pronounced in the low-income and low-education youth groups.

**Discussion:**

The networked characterization of Internet use shapes youth self-reported health through both healthy lifestyles and interpersonal interactions. Under the impact of COVID-19, the online information support pathway of youth self-reported health has declined, while the online emotional support pathway has become more prominent, suggesting a breakthrough of the physical isolation limitations of pandemic-proof social capital. Networked characterizations had a more significant improvement in the self-reported health of disadvantaged youth, suggesting that Internet use may have positive potential to narrow health inequalities.

## 1 Introduction

As the core subjects of “digital natives”, youth have a natural symbiotic relationship with the development of digital technology. This symbiosis is manifested in the fact that Internet technology has profoundly shaped the way of lifestyle and socialization of youth. The networked characterization profoundly shapes the self-reported health of youth through the dual paths of lifestyle and interpersonal interaction. In terms of healthy lifestyle, on the one hand, youth are at a critical stage of study and work, facing the double pressure of heavy academic tasks and work competition, forcing them to speed up the pace of life. While youth are growing up in the digital age of rapid development of Internet technology. Mobile smartphones bring convenient life services such as mobile payment, online shopping, takeaway delivery, online car rental, etc., which greatly improve the efficiency of life, help youth to save more time and energy. This alleviates the psychological pressure of youth's studies and careers to a certain extent, which forms a strong demand for a network-driven and efficient lifestyle. On the other hand, healthy lifestyles consist of diet and exercise. Compared with older generations who pay more attention to traditional diets, youth are more willing to try diversified healthy diets, such as light diets and rely more on Internet information to guide their diets. In addition, older generations tend to prefer more traditional forms of exercise, such as morning exercise, square dance, etc., and the purpose of exercise is mainly to strengthen the body, while youth are more in pursuit of challenging and fashionable exercise, such as fitness. At the same time, youth pay attention to the professionalism of the exercise equipment, and they often use smart health devices to monitor exercise data. This has contributed to the development of a networked lifestyle that supports youth health through accessible information.

In terms of interpersonal interaction, youth in the digital age have a strong social need and frequent online interaction behaviors driven by them, reflecting the special characterizations of youth’s ability and frequency of Internet use. As for Internet use ability, youth are relatively proficient in mastering and utilizing various digital social tools, and quickly adapt to new social applications and functions. As for the frequency of Internet use, youth use fragmented time to interact anytime and anywhere, such as online socializing in the gaps between commuting and classes, and some of them even spend long hours immersed in online socializing at night. This is because, on the one hand, the Internet allows youth to break through geographical constraints and establish “weak-relationship emotional connections” through communities of interest. Although these relationships lack deep trust, the sense of belonging to a group can provide emotional resonance and stage support, such as “shallow socialization” and “hitchhiking socialization” which allow youth to satisfy their social needs as well as obtain emotional resonance. On the other hand, instant messaging tools have strengthened the emotional sustenance of strong relationships over long distances. High-frequency network interactions, such as daily sharing and video “cloud companionship”, allow the emotional support of family and friendship to break through the physical space limitations, create new emotional interaction scenarios, and enhance relationship viscosity. As a result, a healthy youth shaping path of networked emotional support for interpersonal interaction has been formed.

However, the health of youth is facing enormous challenges as the social mode of operation has shifted dramatically during COVID-19, with isolation measures becoming an important means of outbreak prevention and control. The impact of COVID-19 on youth health is not only limited to viral infection, but also has long-term effects through the path of changes in lifestyle and socialization. During this particular period, youth’s learning, work, life and socialization have suffered unprecedented impacts, and the networked impact of Internet use on youth’s self-reported health has also undergone important changes. In terms of lifestyle, during COVID-19, the average daily Internet use time of youth rose overall, and was concentrated on entertainment and social content, with a smaller proportion used for sports-related content, suggesting that the fragmented use pattern of Internet entertainment crowded out youth time for physical exercise. This “attention hijacking” leads to a decrease in the frequency of regular exercise among youth who use the Internet more frequently. In addition, in the context of physical isolation that reduces youth’s outdoor activities, the static posture associated with Internet use replaces dynamic physical exercise for a long time, increasing youth’s sedentary time and habits, which leads to an increase in the prevalence of shoulder, neck, waist, and leg pain as well as obesity. This ultimately brings about a diminishing effect of online exercise support for youth self-reported health.

Physical isolation during COVID-19 and control has given rise to the “immediate need” for online socialization among youth, and instant messaging tools have “seamlessly replaced” offline socialization. Internet has given rise to a large number of spontaneous psychological support groups, such as “COVID-19 anxiety mutual aid group”, constituting a new model of emotional mutual aid, and the proportion of youth seeking emotional support in online communities has risen significantly. The “immersive” interaction on short video platforms creates an emotional experience similar to that offline, and this “alternative embodied cognition” enables youth to perceive the “presence” of peers when they are unable to meet offline. Therefore, online socialization during COVID-19 helped youth realize the “digital reconstruction” of emotional support, bringing about an increasing effect of online interaction support for youth self-reported health.

Internet use plays a supportive role on the self-reported health of disadvantaged youth. Disadvantaged youth are often faced with the double dilemma of a lack of economic, educational and social capital resources and the accumulation of health risks (psychological stress, social exclusion). The “low-threshold access” characterizations of Internet, such as the popularization of smartphones and free information platforms, can compensate for the disadvantages of them to a certain extent. On the one hand, the Internet brings universal access to information, and disadvantaged youth can break down geographic and economic barriers and reduce the cost of acquiring health knowledge through online medical platforms and public education resources. On the other hand, the Internet has brought about the reconstruction of social connections. Social media not only provide disadvantaged youth with online emotional support to alleviate their sense of loneliness and alienation, but also expand online socialization to offline interactions, facilitating both online and offline interpersonal interactions. This demonstrates the positive influence of networked characterizations on self-reported health of disadvantaged youth, suggesting the positive potential of Internet use to reduce health inequalities.as well as digital technology in promoting social equity and individual development.

Domestic and international research on the relationship between the Internet and health has broadly developed a dichotomous view. The health promotion theory holds that Internet use has a significant positive effect on the health of individuals (1, 2),and brings health benefits by narrowing the digital divide (3). While the health inhibition theory suggests that Internet addiction is a significant factor in the health inhibition of Internet technology. Prolonged Internet entertainment can negatively affect health by decreasing interpersonal interaction and increasing loneliness and thus. Excessive use of the Internet, as well as reliance on online social media, both increase the health risks of Internet users (4). In recent years, there are also many studies related to the impact of the Internet on health, but in general there are still the following shortcomings: first, in terms of the research object, most of the established studies have focused on the elderly (5–7), rural groups (8), less attention is paid to urban youth groups. While contemporary youth have generally realized Internet access, their lifestyles and interpersonal interactions are highly dependent on Internet use, so it is of great practical significance to pay attention to the impact of Internet use on self-reported health among urban and rural youths. Second, existing studies have not yet placed the analysis of the mechanism of the Internet’s influence on youth self-reported health in the particular scenario of COVID-19. For example, some scholars believe that the leisure and entertainment functions of social use of Internet have a positive impact on the enhancement of youth mental health (9). The “hyper-network” characterization of digital technology expands the boundaries of interpersonal interaction (10); the “clustered” characterization (11) of Internet communication has a positive effect on mental health through social support. At the same time, some scholars have suggested that Internet use has a “double-edged sword effect” on youth health, and have included the mediating factors of social network as well as health management in the explanatory framework of “technology-health” (12). However, none of the above studies considered whether the impact of COVID-19 on the support of interpersonal interactions and healthy lifestyles of youth had changed. Because for the youth group who have already adapted to the fast-paced life, the sudden COVID-19 seems to press the pause button in the rapid development stage, and the youth’s life pattern and interaction mode will inevitably be affected. As Internet is the primary medium through which youth engage with the outside, it is more relevant to discuss its impact on youth's self-reported health during COVID-19.

Based on the above shortcomings, this paper attempts to conduct empirical analysis using CGSS2017 and CGSS2021 data. The Chinese General Social Survey is a nationwide, comprehensive, and continuous academic survey project conducted by the China Survey and Data Center at Renmin University of China. The survey was scientifically sampled, had a large sample size, and was highly representative, ensuring the reliability of the research conclusions. Both CGSS2017 and CGSS2021 cover information related to this research institute's focus areas, including self-reported health, lifestyle, interpersonal interaction, and internet use among youth. Furthermore, CGSS2021 is the most recent database available in publicly accessible data. In addition, CGSS2017 and CGSS2021 represent the years before and after COVID-19, respectively, which enhances the validity of the data analysis in this study. On the basis of the existing studies, this paper focuses on whether and how the impact of Internet use on youth self-reported health is affected by COVID-19. This is intended to further expand the contextual boundaries of the “Technology-Health” analytic framework, which has important theoretical value for the healthy development of youth.

## 2 Digital health support framework during COVID-19

### 2.1 Digital support system for youth self-reported health

**Figure 1:**
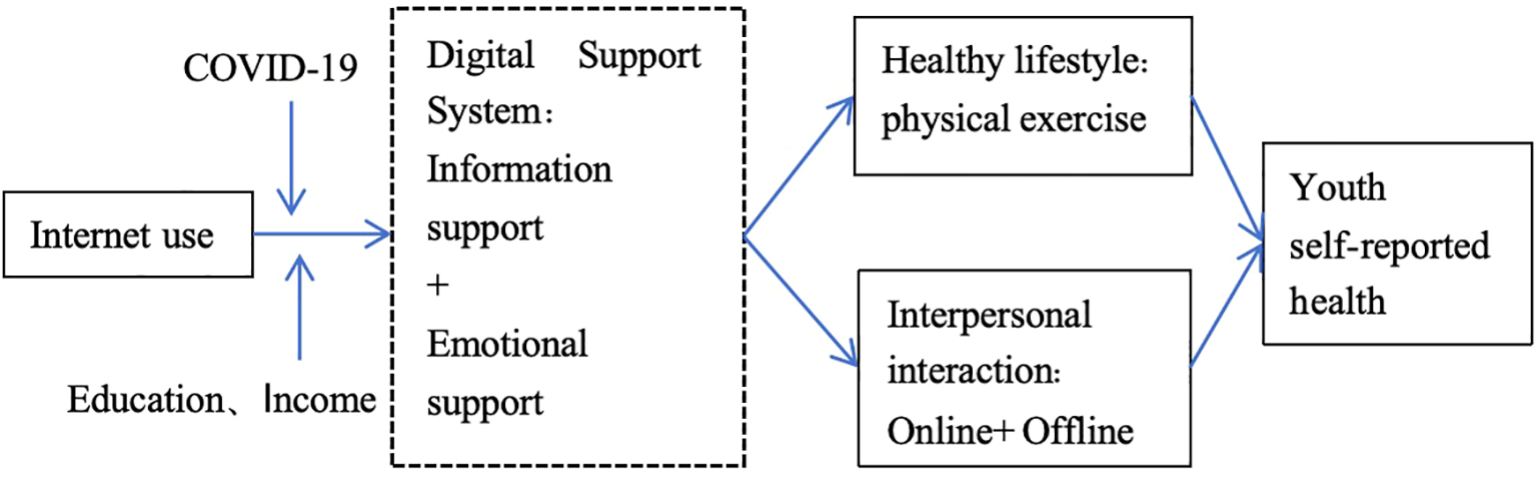
The role of digital support in youth self-reported health over the course of COVID-19.

In the research on how Internet use affects health, there are two main paths: one is the information acquisition mechanism. In the network society, as a communication medium, Internet is an important source of health information. Internet users can find health knowledge, search for information on disease characteristics, enhance health prevention and care, participate in online health activities, improve lifestyle and other activities to improve individual health (13). The second is interpersonal emotional mechanisms, including social participation, social activities, leisure and entertainment, etc., mostly focusing on explanations of mental health (14). This results in the formation of two functions that Internet technical support has, namely the function of information support and emotional support. Technical support is an important form of social support, this paper further expands from the social support perspective to analyze how Internet use can promote youth self-reported health by providing information support for lifestyles and emotional support for interpersonal interactions, respectively. The analytical framework is shown in **Figure 1**.

First, the information dissemination function of Internet is relevant to youth healthy lives. The Internet provides youth people with scientific knowledge of exercise physiology, personalized programs and injury prevention guidelines, so as to reduce the risk of blind exercise. Using smart bracelets to help youth develop healthy exercise and dietary habits, focusing on health data synchronization and dietary calorie calculation. Transforming abstract “health goals” into quantifiable behavioral indicators can help youth understand the necessity of exercising through cognitive enlightenment and help them clarify “how to exercise” and “what to do” through action anchoring. By reducing the “knowledge blind spot” and “implementation barrier”, these networked information supports make it easier for youth to transform their health knowledge into regular exercise behaviors, maintain a healthy lifestyle, and ultimately improve their self-reported health. At the physiological level, aerobic exercise improves cardiorespiratory fitness and strength training increases muscle mass, which directly improves the perception of physical function. At the psychological level, exercise triggers the secretion of endorphins to alleviate depression and anxiety, and at the same time improves self-efficacy through the “self-determination theory”. Therefore, the networked information support empowers youth’s self-reported health by increasing the frequency of physical exercise.

Secondly, the emotional connection function of Internet is related to the interpersonal interaction of youth. The likes and comments on WeChat’s circle of friends and the interactions on late-night public websites satisfy youth’s emotional needs for attention and understanding. In the context of rapid urbanization and marketization in China, youth often leave their hometowns and go to the cities alone to pursue education and employment, practicing what Baumann refers to as “fluid modernity”, i.e., the original mode of social relations is no longer binding, and interpersonal connections tend to break down (15). Frequent population mobility has led to the gradual decline of geo-relationships and the alienation of interpersonal relationships in cities. The traditional “society of acquaintances” has been transformed into “society of strangers” with a tendency for interpersonal interactions to become individualized (16). Interest groups, game groups, and other online communities form a sense of communion through common topics, which can help alleviate the atomized loneliness of youth brought by urbanization. In addition, the experience sharing of Zhihu’s “Newcomers to the Workplace” and the resource linking of Rednote’s “Exam Preparation” mutual support community combine emotional support with practical problem solving. This help youth reduce social anxiety, expand their social radius, and promote more active participation in real-life interpersonal interactions. Therefore, networked emotional support may help youth promote interpersonal interactions and thus empower their self-reported health.

### 2.2 Impact of COVID-19 on youth digital support systems

COVID-19 has reshaped the trajectory of youth’s lives by restricting their ways of socializing and living through mandatory physical segregation. In the tense relationship between the closure of physical space and the extension of digital space, both the vulnerability of traditional offline modes of interaction are exposed and adaptive survival strategies based on Internet use are constructed. At the same time, this particular historical situation also provides a key natural experimental field for us to observe the transformation of socialization mode and life mode of youth embedded in digital technology. Some scholars proposed the concept of "virus-combat social capital" to examine the changes in social connectivity during COVID-19. The virus-combat social capital can be maintained and increased by strengthening the closeness of relationships and increasing online interactions even when interactions in the physical space are hindered (17). On this basis, this paper further examines the impact of networking on youth lifestyle and interpersonal interactions during COVID-19. Physical isolation made the online dissemination of information and emotional functions particularly important. Internet use during COVID-19 serves as both an “information beacon” and an emotional buffer for youth, building a digital support system for them to prevent COVID-19. However, Internet use during COVID-19 was limited in its information support for physical exercise due to the disconnection of offline scenarios, but played a key role in its emotional support for interpersonal interactions, filling the interpersonal interaction “vacuum”.

#### 2.2.1 Decline in the information support function of physical exercise

After the outbreak of COVID-19, space control and restrictive policies led to the closure of public sports venues such as gyms and stadiums, so that youth lost the public physical space for regular exercise, and group sports were limited by social distance requirements, so that the public basis for sports participation was missing, and the community connection function of offline collective activities was blocked. It is difficult for youth to build a consensual sports cultural identity which will affect the fulfillment of the needs for self-identification. In addition, the average daily Internet use time of youth during COVID-19 surged, including online classes, work, entertainment, and socialization. The proportion of sedentary behavior rose, compressing the time for physical exercise. Platform algorithms prioritize short videos, games, and other content. The longer youth spend screen time on such content, the more likely they are to form an "information cocoon" and reduce the reach of exercise information. There is a clear correlation between increased screen time and decreased physical exercise among youth. COVID-19 has exacerbated the problem of sedentary youth, which is seen as a microcosm of the youth health crisis. The uncertainty associated with COVID-19 increased negative emotions such as anxiety and depression, which directly reduced youth motivation to exercise, and in turn, physical exercise behaviors. Therefore, although Internet use provides rich information support (e.g., fitness tutorials, health science) and increases the frequency of physical exercise among youth (18), but under the influence of COVID-19, the information support function of networked in exercising physical fitness is weakened, further challenging the physical and mental health development of youth.

#### 2.2.2 Emotional support function enhancement in interpersonal interactions

As “digital natives”, youth have a strong advantage in the use of the Internet, and demonstrate greater flexibility and adaptability in their interpersonal interactions. This characteristic is manifested in the special situation of COVID-19, as they quickly shift from offline socialization to online socialization and maintain their social activity. Active online socialization also brings rich emotional support to youth, including game socialization, which provides virtual coexistence experience and has a certain alternative function to offline socialization; short video and live broadcasting interaction creates a new way of social expression and enhances emotional connection. Anonymous community provides a channel for psychological pressure to be ventilated, and reduces the sense of loneliness. For youth, online socialization during COVID-19 was dominated by weak connections, such as interest communities and industry exchange groups, in which youth shared their COVID-19 experiences, moods and confusions with others, and also obtained comfort, advice and encouragement from these virtual communities. The emotional interactions and support in these virtual communities provide youth with a channel for emotional sustenance and catharsis, and have become an important source of emotional support for youth. This may help youth alleviate their loneliness and psychological pressure during the quarantine period, and promote mental health development. It is similar to the function of “virus-combat social capital” to alleviate the loneliness brought by home isolation, but also different from the strong relational connection characteristic of “virus-combat social capital” (17).

The resilience of youth’s online social networks determines that their interpersonal interactions will be less affected by COVID-19. Although physical isolation blocks offline communication opportunities, youth can still maintain and expand their social networks through online socialization. This represents youth's unique “digital social immunity” function. In other words, by reconstructing interpersonal interaction and expanding social connections, online social networking builds a new type of interpersonal support system during the period of physical isolation, thus enhancing the adaptive capacity of youth in risky societies. It plays an important positive role in maintaining the healthy psychological state of youth during isolation.

### 2.3 Heterogeneity of youth self-reported health

Health stratification studies have found that people’s education and income status are closely related to their health status (19, 20). The “social causation theory of health” (also known as the "structural theory of health") considers differences in social resources determined by socioeconomic status, such as education and income, to be an important source of health inequality. Advantageous groups, represented by middle and high socioeconomic status, have easier access to health resources for health protection, while disadvantaged groups will systematically face more health risks as well as diseases and present poorer health status (21). For example, those with advanced degrees are more advantaged in terms of health care knowledge acquisition and utilization of medical information and technology (22), leading to healthier lifestyles (23). Higher-income individuals are empowered to be healthy through access to better material conditions, such as diet, living environment, health insurance, and healthcare expenditures. However, when exploring the relationship between socioeconomic status and health inequality, the traditional theory of social stratification represented by social causation theory ignores the changes in social structure and information flow methods during the transition from traditional industrial society to digital society, as well as the impact of the development of digital technology on health inequality (24). Along with the change of the power structure of the traditional industrial society in the network society, the way of information and resource flow has also changed. The Internet technology, while promoting the normalization of information distribution and the networkization of information flow, presents diversified knowledge and information to the general public and promotes the realization of the common people’s access to information (25).

As a carrier of information resources, Internetmay modulate the impact of socioeconomic status on youth self-reported health, breaking the dilemma of unmet health needs in traditional societies due to insufficient information resources. This may help to reduce health inequalities (26). In terms of information support for healthy lifestyles, advantaged youth, by virtue of their own structural advantages, possess sufficient material resources and economic support, grasp COVID-19-related information through their own high-quality social networks, and maintain good healthy lifestyles. When advantaged youth have health problems, they can also enjoy better medical services (27), obtaining better health returns. In this case, the informational support provided by Internet use to advantaged youth is dissipated or replaced by their own status resources, which in turn have limited positive effects on health. In contrast, for disadvantaged youth with less education and lower economic incomes, Internet use can break through the constraints of educational and income deficits and serve the remarkable function of networked information support. In terms of emotional support for interpersonal interactions, advantaged youth usually have richer real-world social networks, including family support, occupational circles, and high-quality social resources from their educational background. These offline supports are sufficient to meet the emotional needs of advantaged youth such as confiding, recognition, and sense of belonging. Internet serves more as a channel for instrumentalization rather than emotional dependence, so the marginal benefit of Internet use in promoting the self-reported health of advantaged youth is low. For disadvantaged youth with low educational attainment and low income, who tend to have weaker offline support networks, e.g., family economic pressures and restricted social circles, the Internet has instead become a core channel for them to expand their interpersonal interactions and obtain emotional support. The low-cost social connections brought about by the use of the Internet build a no-threshold social network for disadvantaged youth. The popularized emotional expression, localized mutual support network and psychological empowerment form an emotional compensation mechanism to alleviate the loneliness and anxiety of them. This may enhance disadvantaged youth's psychological resilience, which in turn has a better self-reported of health and positive effects.

## 3 Research hypothesis

### 3.1 Dual empowerment pathways of digital support systems for youth self-reported health

The Internet provides young users with information support based on a multifaceted health information search knowledge base. Youth who access health information resources based on the Internet are more likely to develop a healthy lifestyle and tend to adopt a series of behavioral patterns that maintain and promote health (28). On the one hand, the new mode of “Internet + fitness” is becoming more and more common. Short videos, live broadcasting platforms and online sharing platforms have enhanced the enthusiasm and initiative of youth to participate in physical exercise (29). On the other hand, based on Internet information access, youth can both understand the benefits of scientific exercise and healthy sleep sleep. At the same time, they are able to reduce or avoid misconceptions about exercise and sleep (30), thus showing the positive effect of Internet use on health behavior habits. Therefore, the health information support provided by the Internet for young users leads to higher levels of health through the maintenance of healthy lifestyles.

The Internet brings emotional support to youth’s interpersonal interactions using social media as a technological vehicle. On the one hand, youth who are good at using the Internet have stronger initiative in the practice of social media. The Internet builds a virtual space for youth interpersonal interaction, and the popularization of social platform applications satisfies the emotional sharing needs of young users, specifically including expression and sharing, social identity, emotional companionship, self-exploration, and intimacy. On the other hand, based on the developmental needs of youth in the stage of schooling or work, online socialization has become an important way to improve the quality of social capital through the capital transformation mechanism of emotional support. The emotional support provided by Internet applications such as anonymous communities, instant messaging and AI accompaniment is exactly the process of accumulating “symbolic capital” proposed by Bourdieu (31). This process allows the emotional value that youth gain in online empathy to be transformed into a foundation of trust for weak online connections. Network socialization expands youth’s interactions with weak relations and strangers, promotes the improvement of social network structure. It also improves the heterogeneity, extensiveness, and attainment of social capital, and then enriches the content of social network resources. It is conducive to the mobilization of social capital and realizes the virtual-real conversion of social capital (32), ultimately forming high-quality social capital of youth. High-quality social capital can be used as a filter for youth’s emotional support. For example, it can help youth establish emotional connections in technical interactions in professional forums, forming a positive cycle of ability recognition-emotional satisfaction-capital appreciation. Therefore, youth who expand their interpersonal interactions based on the Internet have the opportunity to improve the quality of their social capital, obtain more emotional support, and enhance their self-identity, which in turn leads to a higher level of self-reported health.

In short, the informational and emotional support that Internet use brings to youth significantly contributes to youth’s self-reported health by promoting physical exercise and interpersonal interactions, respectively. Accordingly, the two research hypotheses of proposition one are formulated.

Hypothesis 1.1: Frequency of Internet use enhances self-reported health among youth through increased physical exercise.

Hypothesis 1.2: Frequency of Internet use enhances youth self-reported health by facilitating interpersonal interactions.

### 3.2 COVID-19, digital support systems and youth self-reported health

On the one hand, there are the following main interpretations of the idea of “declining self-reported health of youth in 2017-2021”. Digital Alienation Theory argues that between 2017-2021, the average daily use of smartphones by youth increased, and this screen dependence caused increasing health problems, which in turn reduced self-reported health. The economic stress theory suggests that the slowdown in our economy since 2018 and the rise in youth unemployment has led to their existential anxiety eroding health perceptions, which in turn reduces self-reported health. Epidemic Dominance Theory argues that epidemiological policies lead to social isolation, which has a negative impact on the mental health of youth. In addition, the persistence of COVID-19 among those infected with NKP manifests itself in weakened physical functioning such as fatigue and muscle pain, exacerbating the negative expectations of youth health and, in turn, lowering self-reported levels of health.

On the other hand, there are the following main explanations for the view that “youth self-reported health is on the rise from 2017-2021”. Digital Support Theory argues that online emotional confabulation reduces the risk of depression in youth, which in turn enhances their self-reported health. Structural Improvement Theory holds that education improves the health perceptions of highly educated youth through increased health literacy and access to resources, which in turn improves self-reported health. Economic security reduces survival anxiety among high-income youth, which in turn enhances self-reported health. Occupational stability has a protective effect on youth mental health, which in turn enhances self-reported health.

While the above explanations provide possible perspectives on influencing youth's self-reported health, they are still insufficient in analyzing the mechanisms by which Internet use affects youth's self-reported health. Most of the existing explanations are based on single-factor, static, and macro-micro split explanations, ignoring the fact that self-reported health is the result of the action of multiple social factors, thus leaving many unexplained differences in changes, and failing to provide reasonable explanations for the dynamic trends in self-reported health among youth. In this paper, we will analyze the multifaceted factors affecting youth's self-reported health over time and in a comprehensive manner by constructing a multidimensional research framework. Combining macro- and micro-mechanisms, this paper attempts to explore the impact of COVID-19 at the macro level and Internet use at the individual level on the self-reported health of youth, in an attempt to establish a complete logical structure that encompasses the macro- and micro-results. Because the isolation precautions brought about by COVID-19 increased the number of hours of individual Internet use, this is a prerequisite for changes in youth self-reported health. Therefore, it is important not to ignore the time-conditioned effects of macro public health events based on micro-mechanisms affecting youth's self-reported health.

During physical segregation, offline fitness venues are closed and outdoor activities are limited, challenging youth to maintain a healthy lifestyle through physical exercise. Although the Internet can bring certain resources and pathways for youth’s online exercise, the effect of promoting youth’s health has certain limitations. Because youth attach particular importance to the construction of self-identity in physical exercise, this feature reflects the strong social identity needs of the youth group, which makes them pay more attention to the “social display” function of offline physical exercise, and strengthen their self-identity through the recognition of fitness effects by peers and professional coaches. During the period of physical isolation, “digital fitness” cannot satisfy this special need of youth, resulting in a significant decline in the their motivation to exercise. In addition, due to the transitional characteristics of the life stage of youth, COVID-19 brought about by the interruption of education, career development uncertainty has exacerbated the vulnerability of the transitional stage of youth, so that they are facing greater academic and occupational pressures. Physical exercise at this particular time is of limited effectiveness in reducing stress in youth who have a tendency to “have a good time”. They turn more to online games or short video apps, making it difficult to maintain a healthy lifestyle through online exercise patterns.

**Figure 2:**
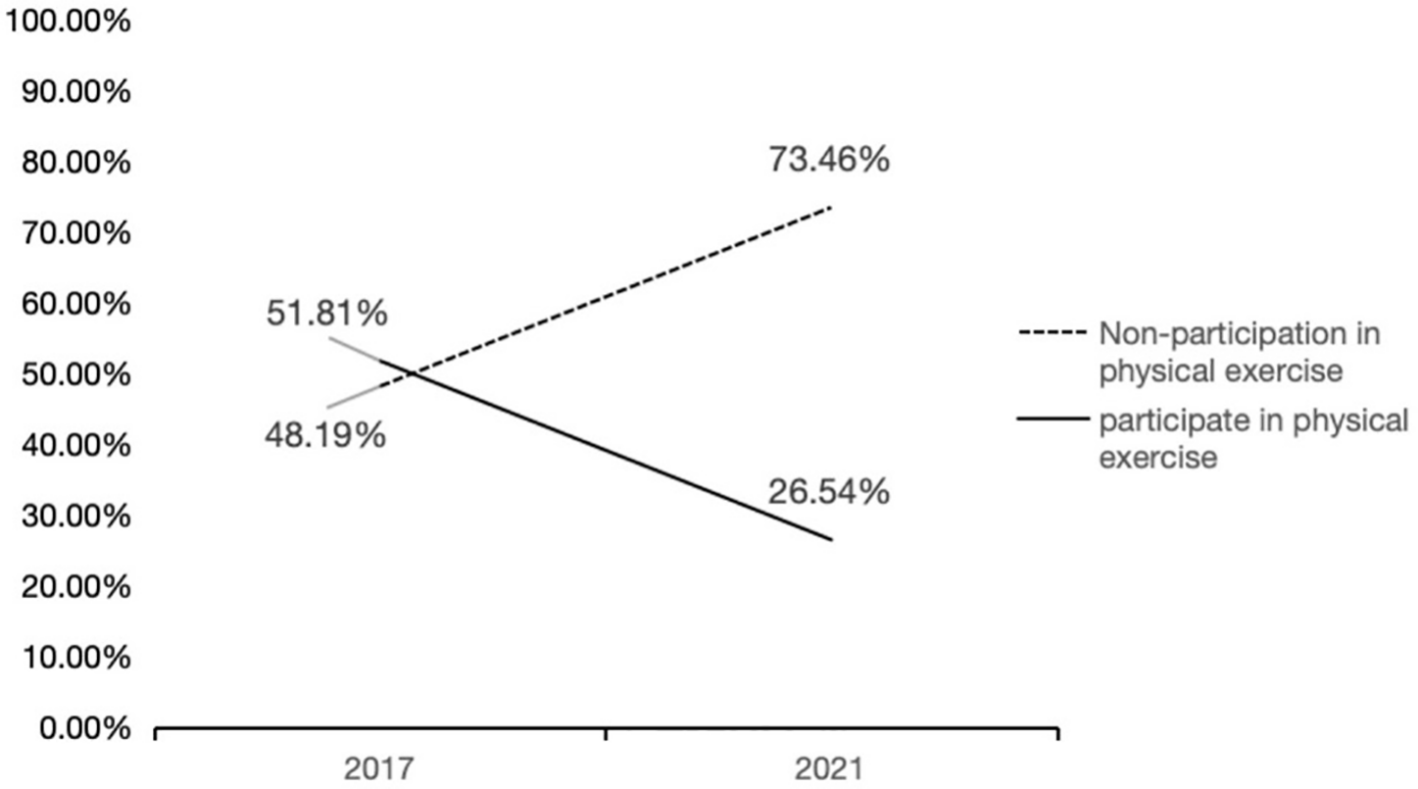
Trends in physical exercise among youth over the course of COVID-19 (2017-2021).

COVID-19 has had a significant impact on the physical exercise behaviors of youth. In July 2020, researchers such as Qin Fei of Jinan University and the Scientific Research Institute of the State General Administration of Sport found that nearly 60% of Chinese adults were physically inactive during the early stages of COVID-19, with an average of more than four hours of screen time per day at home, with the longest screen time among youth between the ages of 20 and 24 years old (33). Meanwhile, according to the 2017 vs. 2021 CGSS data in this paper, as shown in **Figure 2**, about 52% of youth were physically active in 2017, while that percentage dropped to 26.54% in 2021. In contrast, in 2017, 48% of youth were not physically active, while this increased to 73% in 2021. This indicates a significant decrease in youth physical exercise participation after the pandemic compared to pre-pandemic period.

In short, the positive effect of the Internet to significantly improve youth self-reported health through physical activity declined under the influence of COVID-19. Accordingly, the research hypothesis of proposition two is formulated.

Hypothesis 2.1: In 2021, the positive effect of Internet use on physical exercise is significantly reduced, as is the positive effect of physical exercise on self-reported health.

Although physical isolation has blocked opportunities for offline interaction, it has given rise to a demand for online socialization among youth, who can still maintain and expand their social networks through online socialization, presenting unique “digital interaction immunity” function. The resilience of online socialization determines that the interpersonal interactions of youth are less affected by COVID-19. During isolation, the average daily messages on real-time chatting tools increased, which became the main channel of their emotional connection. By reconstructing the way of interpersonal interaction, innovating the way of communication, and expanding social connections, online socialization builds a new type of interpersonal support system during physical isolation, thus enhancing the adaptive capacity of youth in a risky society. It plays an important positive role in maintaining a healthy psychological state of youth during COVID-19.

Internet technologies such as video calls and voice linking build a sense of “being together”, enabling youth to feel accompanied even in physical isolation. Synchronized activities such as watching live broadcasts and online games build a sense of collective participation, providing youth in isolation with the emotional value of “being together”. COVID-19 has diversified the emotional support function of the Internet. Compared to text-only conversations, digital communication methods such as dynamic emoticons, voice messages, and video calls have realized relatively accurate emotional transmission during physical isolation. Youth find like-minded partners through online communities, forming an emotional support network that helps to alleviate youth’s repressed emotions, which in turn has a more pronounced role in promoting youth self-reported health.

In short, the emotional support gained by relying on online interactions during COVID-19 may help to overcome the limitations on interpersonal interactions imposed by COVID-19 and physical isolation to further promote youth’s self-reported health. In other words, the positive effect of Internet use may help to enhance youth self-reported health through interpersonal interactions rose under the influence of COVID-19. Accordingly, the alternative research hypothesis of proposition two is proposed.

Hypothesis 2.2: In 2021, the positive impact of Internet use on social interactions is significantly greater, as is the positive impact of social interactions on self-reported health.

### 3.3 Heterogeneity of youth self-reported health

There was a significant class differentiation in people’s mental health levels during COVID-19, that is say, lower socioeconomic status brought higher levels of depression for individuals (34). Does the health stratification effect under COVID-19 also exist among youth? Is the positive influence of social support functions brought about by Internet use on youth health affected by COVID-19? With the development of our digital infrastructure and widespread Internet access among youth, the growth of Internet users has changed the pattern of health information distribution (35). In this context, the positive effect on health brought by the Internet during COVID-19 was limited for advantaged youth with higher levels of education and income. The support of lifestyle information was specifically demonstrated in the following ways: first, Internet use breaks down geographic and economic constraints, lowers the threshold of access to epidemic prevention information for disadvantaged youth. The internet allows low-income youth to access epidemic prevention and health information without paying extra, reducing the risk of COVID-19 due to information asymmetry. It differs from the health inequality outcomes of the digital divide for older adults (36). Low-education and low-income youth can use official media, social media, government websites and other channels on the Internet to obtain timely information about epidemic dynamics, prevention and control knowledge, policy measures, etc. They protect themselves and reduce the risk of infection by learning about the development of the epidemic, common epidemic prevention and treatment methods. Second, disadvantaged youth can use Internet channels such as e-commerce platforms and community group purchases to obtain information on the procurement of living materials and secure their basic needs. Third, the Internet provides abundant employment information resources. Low-education and low-income youth can obtain information about job positions, part-time job opportunities and business start-up projects through job websites, social media groups and other platforms, which help them maintain their income or find new sources of income during COVID-19. Fourth, Internet service software helped disadvantaged youth manage their health remotely during COVID-19. For example, Alipay’s “Healthcare” module provided BMI calculations and medication reminders, partially replacing high-priced private doctor services. Fifth, in terms of physical exercise, Keep has launched a “zero-based fitness plan”, using an achievement system to motivate low-income youth to persist in physical exercise during COVID-19. They can join free exercise activities and earn points for punching cards to increase the frequency of physical exercise during the physical quarantine. Based on the above information support, disadvantaged youth can break down knowledge barriers to health information access through high-frequency Internet use during COVID-19, realize health information inclusion, compensate for the lack of health knowledge that may be caused by a lower level of education, and still maintain a healthy lifestyle during COVID-19.

Emotional support for interpersonal interactions is manifested in the following ways: first, through low-threshold tools such as WeChat voice or video, disadvantaged youth are able to maintain high-frequency contact with their families and hometowns across the border. Video calls have become a major means of “cloud reunions”, especially for young migrant workers, to reduce the loneliness that results from isolation. For example, many low-income delivery riders find emotional solace between jobs by sharing their daily routine with their families through WeChat groups. Family groups and hometown groups have become the core carriers of emotional support. Sharing the news of COVID-19 in their hometowns and advising each other on the protection against the epidemic can strengthen the sense of belonging to the group, so as to avoid the sense of "drifting" due to the city's sealing control. Second, in Tik Tok live rooms or fan groups, low-educated youth find identity by commenting to participate in communities of interest and interacting with peer groups. For example, unemployed low-income youth who joined the “home-based side hustle exchange group” not only obtained income-generating information, but also gained “peer-to-peer emotional support” by complaining about their stress and sharing their daily life chores in the group. Third, game socialization, such as the King of Glory team, has become a channel for disadvantaged youth to release pressure. The sense of achievement in virtual collaboration to alleviate the pressure and anxiety brought by COVID-19, especially for the disadvantaged youth who lack online social resources. The network community has become the “second social circle” and the space for the expression of emotions, which promoted the emotional support of disadvantaged youth. Because in online socialization, people pay more attention to the congenial of interests and views, while the influence of education and economic income is relatively small. The Internet provides a variety of virtual social scenarios. Low-educationand low-income youth can portray multiple roles in these forums, communities, and online game scenarios, display their interests, talents, or personalities, and interact with others. Such virtual identities are not constrained by their real identities, which helps disadvantaged youth establish a wide range of social relationships during COVID-19, and expand the circle of interpersonal relationships. On the basis of obtaining identity, disadvantaged youth may extend this social relationship to real life after COVID-19. This may lay the foundation for offline interactions in the post-epidemic life, in order to obtain more social support and resources, and make up for the lack of information and emotional support in the real social circle. Based on the above emotional support, disadvantaged youth may break isolation through the Internet social platform, build emotional communities, and construct social support networks for themselves through the form of online mutual assistance. Therefore, Internet use may serve as a lever for disadvantaged youth to improve their self-reported health, helping them reap more health dividends through high-frequency Internet use.

In short, Internet use may have a positive impact on disadvantaged youth's self-reported health. This view may bridge health inequalities in the Social Causality Theory to some extent. In other words, Internet use has better health-enhancing effects on disadvantaged youth with lower economic income and education. Accordingly, the two research hypotheses of proposition three are formulated.

Hypothesis 3.1: The lower the income level of youth, the greater the enhancement effect of frequency of Internet use on their self-reported health.

Hypothesis 3.2: The lower the level of education, the greater the enhancement effect of the frequency of Internet use on the self-reported health of youth.

## 4 Research design

### 4.1 Data sources

The data used in this paper comes from the Chinese General Social Survey (CGSS), which is the first national, comprehensive and continuous academic survey project in China. CGSS is executed by the Survey and Data Center of Renmin University of China. The survey has scientific sampling, large sample size and strong representation, which can better guarantee the reliability of the research findings. Considering the time point of the outbreak of COVID-19 and the availability of data, this paper selects the data of CGSS2017 and CGSS2021. According to the needs of the study, this paper screens and retains the samples of the youth group (18–45 years old), and the total sample size is 6907 after data cleaning.

### 4.2 Selection of variables

Based on the age definition of youth, only the 18-45 cohort is retained in this paper. In the income sample, in order to avoid the influence of extreme values, income above the millionth percentile and missing values of “not applicable”, “don't know”, and “refused to answer” were deleted. In the Internet use, self-assessed health, and mental health samples, missing values for “don't know” and “refused to answer” were removed. The dependent variables in this study measured healthy lifestyle, interpersonal interaction and self-reported health.

Healthy Lifestyle. The 2017 questionnaire asked “In the past 12 months, how many times a week did you perform physical exercise that lasted up to 30 minutes and would have made you sweat, on a typical day?”. The options “0 times” and “less than once” were combined and coded as “0” to indicate non-participation in physical activity. Code the remaining option as “1” to indicate participation in physical exercise. Remove missing values for “never exercise”, “don't know” and “refused to answer”. The 2021 questionnaire asked “Do you regularly do at least 20 minutes of physical activity that makes you sweat or breathe faster?”. Coding “never” as ‘0’ indicates no participation in physical exercise, and coding the remaining options as “1” indicates participation in physical exercise. Remove missing values for “unable to select” and “refused to answer”.

Interpersonal interaction. The 2017 & 2021 questionnaire asked “In the past year, have you socialized regularly in your free time?”. The options “Never”, “Rarely”, “Sometimes”, ‘Often’, "Very Frequent“ were coded as ”1-5". Larger values indicate a higher frequency of interpersonal interactions. Remove missing values for “don't know” and “refused to answer”.

Self-reported health. The 2017 & 2021 questionnaire asked “What do you feel is your current state of physical health?”. The options “very unhealthy”, “relatively unhealthy”, ‘fair’, “relatively healthy” and “very healthy” were coded as “1-5” respectively. Larger values indicate higher levels of self-reported health. Remove missing values for “don't know” and “refused to answer”.

The independent variable in this paper is Internet use. The 2017 & 2021 questionnaire asked “In the past year, have you often gone online in your free time?”. The options “never”, “several times a year or less”, “several times a month”, “several times a week” and “daily” were coded as “1-5” respectively. Larger values indicate more frequent Internet use. Remove missing values for “don't know” and “refused to answer”.

The main control variables include gender, years of education, log income, marital status, mental health, and year. Gender: Code 0 for females and 1 for males. Education years: The “highest level of education” in the questionnaire was converted to “years of education”. Code “no education” as 0. Code “Private School” as 1. Code “Elementary School” as 6. Code “Junior High School” as 9 . Combine “Vocational High Schools, General High Schools, Junior Colleges and Technical Schools” into 12 . Combine “College and Undergraduate” to code 15 . Code “graduate and above” as 19. Logarithm of income: In order to avoid the effects of skewed distribution, the annual income of the investigators was logarithmized. Mental health: “In the past four weeks, how often have you felt depressed or down”, define the option ‘Never’ as “Healthy” and code it as 1. Define the options “Always”, ‘Often’, “Sometimes”, “Rarely” was defined as “unhealthy” and coded as 0. Year: survey data for 2017 was coded as 2017 and survey data for 2021 was coded as 2021.

The descriptive statistics of the relevant variables are shown in **Table 1**.

**Table 1:** Descriptive statistics of main variables.

Variable	Sample	Average	Standard deviation	Minimum	Maximum
Self-reported health	6907	3.968	0.898	1=quite unhealthy	5=quite healthy
Healthy lifestyle	6907	0.411	0.492	0=Non-participation in physical exercise	1=participate in physical exercise
Interpersonal interaction	6907	2.788	0.972	1=never	5=extremely frequent
Frequency of Internet use	6907	4.567	1.062	1=never	5=everyday
Gender	6907	0.461	0.499	0=female	1=male
Educational years	6907	11.627	3.825	0	19
Log of income	6907	8.509	4.311	0	14.509
Marital status	6907	0.694	0.461	0=unaccompanied	1=be partnered
Mental health	6907	3.950	0.948	1=unhealthy	5=healthy
Year	6907	2018.458	1.925	2017	2021

### 4.3 Analytical strategies

First, the data analysis in this study used multiple linear regression models to validate the effects of Internet use, healthy lifestyle, and interpersonal interaction on youth's self-reported health. We used binary logit regression models to validate the effects of Internet use on healthy lifestyle and interpersonal interaction. Second, this study used multiple linear regression models to test the hypothesis of a moderating effect of COVID-19 on the impact of Internet use on youth self-reported health, incorporating an interaction term between year and Internet use variables. Third, this study used multiple linear regression models to test the hypothesis of heterogeneity in Internet use affecting youth self-reported health, incorporating the interaction terms of log income with the Internet use variable and years of education with the Internet use variable, respectively. Finally, considering the selectivity bias of the Internet use variable, this study conducted a robustness test.

## 5 Empirical analysis

### 5.1 Internet use and youth self-reported health

In this paper, we analyzed the effects of Internet use on youth self-reported health, healthy lifestyle and interpersonal interaction using linear regression or logistic regression models. The results are shown in **Table 2**.

**Table 2:** Multiple linear regression and binary logistic regression models for self-reported health, healthy lifestyle, and interpersonal interaction.

	Model 1 Self-reported health	Model 2 Healthy lifestyle	Model 3 Interpersonal interaction
Control variable	included	included	included
Frequency of Internet use	0.086*** (0.010)	0.127*** (0.029)	0.067*** (0.012)
Healthy lifestyle	0.085*** (0.022)	–	–
Interpersonal interaction	0.056*** (0.010)	–	–
Constant term	2.111*** (0.069)	-2.058*** (0.181)	2.244*** (0.076)
R^2^	0.145	0.089	0.009
Sample	6907	6907	6907

Note: Lifestyle and interpersonal interactions have a significant mediating role in the effect of Internet use on youth self-rated health. *** p < 0.001. Coefficients are unstandardized regression coefficients with robust standard errors in parentheses. Control variables include gender, years of education, marital status, log income, mental health, and year.

Model 1 is a multiple linear regression model of youth self-reported health. It can be found that the regression coefficient of Internet use is significantly positive at the 0.001 confidence level after including all control variables. This indicates that the frequency of Internet use has a significant positive effect on the self-reported health of youth. In addition, the regression coefficients of healthy lifestyle and interpersonal interaction are both significantly positive at the 0.001 confidence level, indicating that youth's participation in physical exercise and high frequency of interpersonal interaction have a significant positive impact on their self-reported health. On the basis of Model 1, this paper further explores the mediating effect of healthy lifestyle and interpersonal interaction. the results of Model 2 indicate that Internet use significantly promotes healthy lifestyle among youth, while the results of Model 3 indicate that Internet use significantly promotes interpersonal interaction among youth. Model 1-Model 3 together suggest that the positive influence of Internet use on youth self-reported health is transmitted through the variables of healthy lifestyle and interpersonal interaction. Hypotheses 1.1 and 1.2 were supported respectively.

### 5.2 Impact of COVID-19 on youth self-reported health

In order to explore the effect of COVID-19 on youth self-reported health, based on **Table 2**, this paper incorporates an interaction term between Internet use and year, an interaction term between healthy lifestyle and year, and an interaction term between interpersonal interaction and year, respectively. Model 4 is a binary logistic regression model for healthy lifestyle and Model 5 is a multiple linear regression model for interpersonal interaction. From **Table 3**, it can be found that after controlling for other variables, the main effect of frequency of Internet use on healthy lifestyle is significantly positive, while its interaction effect with year is significantly negative. This indicates that high frequency of Internet use can significantly contribute to healthy lifestyle among youth. However, this positive effect wanes significantly in 2021. The analysis of interpersonal interaction leads to the exact opposite conclusion, that is, high frequency of Internet use can significantly promote interpersonal interaction among youth. This positive effect is even more evident in 2021.

**Table 3:** Binary logistic regression and multiple linear regression models for healthy lifestyle, interpersonal interaction, and self-reported health.

	Model 4 Healthy lifestyle	Model 5 Interpersonal interaction	Model 6 Self-reported health	Model 7 Self-reported health
Control variable	included	included	included	included
Frequency of Internet use	0.163*** (0.031)	0.048*** (0.013)	–	–
Healthy lifestyle	–	–	0.134*** (0.026)	–
Interpersonal interaction	–	–	–	0.046*** (0.013)
Year	-0.057 (0.342)	-0.485*** (0.136)	-0.024 (0.027)	-0.220** (0.063)
Internet use x Year	-0.243** (0.071)	0.097** (0.028)	–	–
Healthy lifestyle x Year	–	–	-0.099* (0.046)	–
Interpersonal interaction x Year	–	–	–	0.049* (0.021)
Constant term	-2.210*** (0.188)	2.320*** (0.079)	2.508*** (0.057)	2.421*** (0.066)
R^2^	0.090	0.011	0.133	0.135
Sample	6907	6907	6907	6907

Note: There were significant year differences in the impact of Internet use on youth self-rated health. *** p < 0.001, ** p < 0.01, * p < 0.05. Coefficients are unstandardized regression coefficients with robust standard errors in parentheses. Control variables include gender, years of education, marital status, log income, and mental health.

Based on Model 4 and Model 5, this paper further explores whether the positive influence of healthy lifestyle and interpersonal interaction on youth self-reported health was affected by COVID-19. The results of Model 6 show that after controlling for other variables, the main effect of healthy lifestyle on self-reported health is significantly positive, while its interaction effect with year is significantly negative. This suggests that healthy lifestyle can significantly contribute to youth self-reported health. However, this positive effect weakened significantly in 2021. The analysis of interpersonal interaction still leads to the exact opposite conclusion. The Model 7 results indicate that high frequency of interpersonal interaction can significantly promote youth self-reported health. This positive effect is even more evident in 2021. It can be inferred that the positive influence of healthy lifestyle and interpersonal interaction on youth self-reported health was significantly influenced by COVID-19. Hypotheses 2.1 and 2.2 were supported respectively.

### 5.3 Heterogeneous effects of Internet use on youth self-reported health

According to the previous theoretical analysis, self-reported health differences resulting from Internet use among youth are influenced by socioeconomic status to some extent. In order to verify this view, this paper incorporates the interaction terms of Internet use and years of education, as well as the interaction term between Internet use and log income, respectively. From **Table 4**, it can be found that after controlling for other variables, the main effect of Internet use frequency on self-reported health is significantly positive, while its interaction effect with years of education is significantly negative. This suggests that high frequency of Internet use can significantly enhance youth self-reported health. However, this positive effect is more pronounced for youth with lower levels of education. A similar conclusion can be drawn from the analysis of income. The positive influence of high frequency of Internet use on health is more pronounced for youth with lower levels of income. It can therefore be inferred that the positive influence of high frequency Internet use on self-reported health is greater for youth with lower socioeconomic status.

**Table 4:** Multiple linear regression models for self-reported health.

	Model 8 Self-reported health	Model 9 Self-reported health	Model 10 Self-reported health
Control variable	included	included	included
Frequency of Internet use	0.086*** (0.010)	0.135*** (0.022)	0.181*** (0.026)
Educational years	0.021*** (0.003)	0.048*** (0.011)	0.043*** (0.011)
Log of income	0.000 (0.002)	0.000 (0.002)	0.034** (0.011)
Internet use x Educational years	–	-0.006* (0.002)	-0.005* (0.002)
Internet use x Log of income	–	–	-0.007** (0.046)
Constant term	2.111*** (0.069)	1.910*** (0.105)	1.700*** (0.123)
R^2^	0.145	0.145	0.147
Sample	6907	6907	6907

Note: Education and income have a significant moderating role in the impact of Internet use on youth self-rated health. *** p < 0.001, ** p < 0.01, * p < 0.05. Coefficients are unstandardized regression coefficients with robust standard errors in parentheses. Control variables include gender, marital status, mental health, year, healthy lifestyle, and interpersonal interactions.

### 5.4 Robustness tests

Youth’s Internet use may not be randomly assigned, however it may be jointly influenced by a number of confounding variables such as personal characteristics and Internet use habits. These variables may affect both youth’s healthy lifestyles and interpersonal relationships. This can lead to difficulties in meeting the premise assumptions of the regression model, creating self-selection bias and endogeneity problems. Therefore, this paper utilizes dual robust estimation based on the propensity value approach to try to mitigate this self-selection bias and further improve the robustness of the model setup while controlling for the effects of confounding variables.

The Dual Robust Estimates results are shown in **Table 5**. Model 11 and Model 12 are doubly robust estimation models that introduce Internet use variables. Frequency of Internet use was primarily measured by the CGSS 2017 & 2021 questionnaire, “In the past year, how often did you access the Internet in your free time?”. The options were assigned a value from 1 to 5 for “Never”, “Yearly or less”, “Monthly”, “Weekly”, “Daily”, with higher scores indicating more frequent Internet use. On the one hand, based on the consideration of the optimal effect of the model design, the frequency of Internet use is treated in three classifications: low, medium and high. On the other hand, high-frequency Internet use has a negative impact on the health of the population. Especially in youth studies, high frequency of game use leads to poorer health (37). Higher use of online socialization leads to higher levels of online addiction 38). In order to distinguish heterogeneity in the impact of different frequencies of Internet use behaviors on youth self-reported health, as well as referring to Guo Wei's method of classifying the frequency of Internet use (12), in this paper, the frequency of Internet use is further categorized into low, medium, and high. Using low-frequency Internet use as a reference. The results of Model 11 show that the average treatment effect for the treatment group with medium-frequency Internet use is 0.111 and significantly positive at the 0.05 confidence level, and the mean treatment effect for the treatment group of high-frequency Internet use was 0.091 and significantly positive at the 0.01 confidence level. This indicates that the positive impact of high-frequency Internet use on healthy lifestyle among youth is robust after controlling for possible self-selective bias, and the robustness of Hypothesis 2.1 was verified. Model 12 results showed that the mean treatment effect for the treatment group with medium-frequency Internet use was 0.185 and was significantly positive at the 0.05 confidence level. The mean treatment effect for the treatment group with high-frequency Internet use was 0.321 and was significantly positive at the 0.001 confidence level. This suggests that the positive effect of medium- to high-frequency Internet use on youth's interpersonal interactions is robust after controlling for possible self-selection bias. The robustness of Hypothesis 2.2 is verified.

**Table 5:** Dual robust estimates.

	Model 11 Healthy lifestyle	Model 12 Interpersonal interaction
Control variable	included	included
mid-frequency Internet use (reference group:low-frequency Internet use)	0.111* (0.045)	0.185* (0.088)
high-frequency Internet use (reference group:low-frequency Internet use)	0.091** (0.027)	0.321*** (0.059)
POmean	0.288*** (0.028)	2.474*** (0.057)
Sample	6907	6907

Note: *** p < 0.001, ** p < 0.01, * p < 0.05. coefficients are treatment group mean treatment effects, robust standard errors in parentheses.

Interpersonal interaction mostly refers to the process of direct, face-to-face social interaction between individuals (39). Some also need to be mediated, such as new types of interpersonal interactions mediated by the Internet (40). Therefore, in the digital age represented by the Internet, interpersonal interaction has two forms of offline socialization and online socialization (41). Based on the reference to Guo's use of offline social network represented by the Worship Network and online social network represented by the WeChat Network to measure social interactions, as well as the consideration of the operationalization of combining two years' data, on the one hand, this paper chose to use a measure common to both years of data, namely,“In the past year, how often did you socialize in your free time?”measuring youth's interpersonal interactions. In the hypotheses of this paper, this measure encompasses both online and offline socialization.

On the other hand, this paper also selects the variable in the CGSS2018 questionnaire that can directly measure the network size of online socialization, i.e., “How many WeChat friends do you have?” as a replacement variable for emotional support for the robustness test. According to the results of the 2018 data test, as shown by model 13 and model 14 in **Table 6**, the frequency of Internet use has a significant positive effect on youth online interactions, and online interactions have a significant positive effect on youth self-reported health. It can be seen that Internet use enhances youth self-reported health through emotional support brought about by online socialization, meaning that the mediating effect of online emotional support also passes the robustness test. This also demonstrates the ability of Internet use to enhance youth self-reported health not only through offline emotional support, but also through online emotional support.

**Table 6:** Binary Logistic Regression and Multiple Linear Regression Models for Online interpersonal interaction and Self-reported health (CGSS2018).

	Model 13 Online interpersonal interaction	Model 14 Self-reported health
Control variable	included	included
Frequency of Internet use	1.411*** (0.093)	–
Online interpersonal interaction	–	0.236** (0.070)
Constant term	-8.395*** (1.039)	2.293*** (0.152)
R2	0.556	0.093
Sample	3350	3350

Note: Online interpersonal interactions have a significant mediating role in the impact of Internet use on youth self-reported health. *** p < 0.001, ** p < 0.01. Coefficients are unstandardized regression coefficients with robust standard errors in parentheses. Control variables include gender, years of education, marital status, log income, and mental health.

## 6 Conclusions and discussion

The exploration of youth self-reported health has important practical and theoretical significance in the digital age. As the core subjects of "digital natives", youth's Internet use behavior affects their self-reported health by shaping their lifestyles and expanding their interpersonal interactions. Internet technology, with its powerful information dissemination and emotional connection functions, provides social support for youth’s lifestyles and interpersonal interactions. This process not only brings about healthy lifestyles and diversified socialization, but also has certain positive influence on youth self-reported health.

However, in the context of COVID-19, health research has not yet responded adequately to the changes in the networked characterization of Internet-shaped lifestyles and interpersonal interactions as well as their impact on youth self-reported health. Youth’s lifestyles and socialization have undergone dramatic shifts in response to COVID-19. Prevention measures such as isolation policies and social distance restrictions brought about by COVID-19 have disrupted the original rhythm of life and social ecology of youth, with far-reaching impacts on their physical and mental health. Therefore, our study is grounded in the impact of Internet use on self-assessed health among youth in the digital age by combining data from the CGSS2017 and CGSS2021 surveys. By examining how the networked shaping of lifestyle and interpersonal interaction by Internet use in the context of COVID-19 affects youth self-reported health, this study has the following specific findings:

First, the networked characterization of lifestyle and interpersonal interaction in the digital age constitutes a digital support system for youth. The information support and emotional support provided by Internet use promote healthy lifestyles and interpersonal interactions, which ultimately lead to improvements in youth self-reported health. In terms of lifestyle, youth who access health information resources and share health information based on Internet use are more likely to develop healthy lifestyles, such as regular physical exercise habits. In terms of interpersonal interaction, Internet use builds a three-dimensional emotional support network for youth through the synergistic effect of “incremental empowerment” of online interactions and “in-depth expansion” of offline interactions. This networked information support and emotional support together constitute a digital support system for youth, which has a significant gain effect on youth self-reported health. This finding is a reasonable attempt to explore the influence mechanism of youth self-reported health in the digital age, and it is also a useful supplement to the “technology-health” analysis paradigm.

Second, under the influence of COVID-19, the enhancing impact of Internet use on youth self-reported health by promoting healthy lifestyles was significantly weakened, but the positive influence of youth self-reported health by expanding interpersonal contacts was significantly enhanced. This finding not only highlights the function of “virus-combat social capital” as an effective social mechanism for maintaining interpersonal ties and the enhancement of physical and mental health (42). It also expands the boundaries of the role of “virus-combat social capital” in promoting self-reported health among youth through healthy lifestyles represented by physical exercise. Physical segregation during COVID-19 not only limited public physical space for youth to exercise and reduced opportunities for group exercise activities. It also increased Internet use time, compressed physical exercise time and decreased individual motivation to exercise. This results in less frequent physical exercise, a sedentary lifestyle, and increased health risks such as obesity, which in turn inhibits youth's self-reported health. In addition, this finding further extends the function of “virus-combat social capital” in the digital age to emotionally support youth health outcomes. It may help youth to alleviate anxiety and increase psychological resilience so as to better cope with life stresses brought about by COVID-19, and then to promote youth self-reported health.

Third, Internet use among youth has differentiated health health-enhancing impacts. For disadvantaged youth with lower incomes and educational attainment, the enhancing impacts of high frequencyInternet use on their self-reported health were more pronounced. This finding suggests the opposite of the social causation theory of health, namely that technical support for the Internet can help disadvantaged youth break down the constraints on health outcomes that are determined by socioeconomic status, such as education and income. It suggests that Internet use may contribute to narrowing health inequalities among youth to some extent. On the one hand, it may be due, to the economic capital substitution effect of advantaged youth. In other words, advantaged youth with more economic capital are able to obtain high-quality dietary and exercise guidance through a rich material base, and enjoy the health dividends brought about by high-quality living environments as well as medical care. On the other hand, it may be due to the cultural capital substitution effect of advantaged youth. That is, advantaged youth with rich cultural capital can not only receive high-quality education and establish correct health concepts to guide healthy behaviors, such as regular medical checkups, good work and rest schedules, but also establish deep and high-quality social relationships through the highly educated circles screened out by the education stratification mechanism. The "health premium" effect of access to high-quality social capital undermines the positive effect of Internet technology support on the self-reported health of advantaged youth.

Fourth, the above findings have practical implications for the empowerment of self-reported health among disadvantaged groups in the digital age. On the one hand, increasing health information sources for low-income and low-education groups based on the information support function of networking is an important entry point for cracking the health dilemma of disadvantaged groups and promoting health equity. Due to the lack of economic resources, limited education level, and significant digital divide, the disadvantaged groups generally face the problems of narrow access to health knowledge, weak information screening ability, and difficulty in transforming health behaviors. Therefore, the accessibility and appropriateness of health information is the key bridge connecting “health knowledge” and “health behavior” of disadvantaged groups. On the other hand, based on the networked emotional support function, increasing the digital emotional support for low-income and low-education groups is an important measure to help disadvantaged groups resist health risks. Disadvantaged groups face higher risks of psychological stress, such as anxiety, depression, and loneliness, due to economic pressures, lack of social capital, and limitations in the real social circle. As a “digital lever” for health equity, online emotional support has the advantage of being low-cost and highly accessible. This may facilitate increased social connectivity, fix “weak relationship” network deficiencies, and fill gaps in real-world support systems. For vulnerable groups, this may help them to build a “buffer zone” of mental health and a “community” of identity. By resolving the emotional impact of “structural stress”, a deeper shift from “emotional empowerment” to “health empowerment” is ultimately realized.

There are still some limitations in this paper that remain to be discussed. First, this paper only discussed the frequency of Internet use, ignoring the differences in the types of Internet use. Future research can further explore the richness of the types of Internet use, for example, distinguishing the differences in Internet use according to the purpose, function, and duration of Internet use, in order to more deeply explore the positive and negative effects of Internet use behaviors on the impact of youth self-reported health, the heterogeneity, and its boundaries. Second, due to the limited availability of data, this paper only measures healthy lifestyle through the indicator of physical exercise, which includes both physical exercise at the individual level and exercise participation at the collective level. Healthy lifestyle is also a multidimensional variable, and in addition to physical exercise, dietary habits and so on are also important measurement dimensions. Therefore, future research can further examine lifestyle variables multidimensionally from the collective level of exercise activities and healthy diets. Third, due to data limitations, this paper fails to measure the COVID-19 directly, instead choosing indicators for the years over the course of COVID-19 as a proxy. On the other hand, COVID-19 has a certain spatial significance in addition to the temporal significance of the event occurrence record. Therefore, future research can try to add more direct indicators to measure the spatial and temporal meanings of COVID-19. For example, future indicators could reference factors such as physical space segregation, changes in interpersonal relationships, changes in time spent on the Internet, frequency of Internet exchanges, and changes in lifestyles during the epidemic, so as to validate the relevant conclusions with more targeted data.

## Data Availability

The datasets presented in this study can be found in online repositories. The names of the repository/repositories and accession number(s) can be found below: http://www.cnsda.org/index.php?r=projects/view&id=65635422 http://www.cnsda.org/index.php?r=site/article&id=180.
